# A Novel Etchant System for Orthodontic Bracket Bonding

**DOI:** 10.1038/s41598-019-45980-9

**Published:** 2019-07-03

**Authors:** A. I. Ibrahim, V. P. Thompson, S. Deb

**Affiliations:** 1 Centre for Oral, Clinical and Translational Sciences, Faculty of Dentistry, Oral & Craniofacial Sciences, King’s College London, London, UK; 2 Department of Orthodontics, College of Dentistry, University of Baghdad, Baghdad, Iraq

**Keywords:** Orthodontics, Biomineralization

## Abstract

Orthodontic treatment is widely used to correct irregular teeth and/or jaw discrepancies to improve oral function and facial aesthetics. However, it is frequently associated with enamel damage that include chipping, demineralisation, and white spot formation. So far, current bonding systems that can maintain shear bond strengths (SBS) suitable for clinical performance are unable to limit enamel demineralisation, adhesive remnants and damage caused on removal of brackets after treatment. This study reports a novel “safe enamel etch” clinically viable procedure that was accomplished via application of novel etchant pastes developed with β-tricalcium phosphate and monocalcium phosphate monohydrate powders mixed with citric acid (5 M) or phosphoric acid (37% PA) to yield BCA and BPA etchants respectively. Although enamel etched with clinically used PA gel yielded higher SBS than the BCA/BPA etchants, it exhibited greater adhesive remnants with evidence of enamel damage. In contrast, the experimental etchants resulted in unblemished enamel surfaces with zero or minimal adhesive residue and clinically acceptable SBS. Furthermore, the BPA etchant caused lower enamel decalcification with extensive calcium-phosphate precipitation. The study conclusively showed that BPA facilitated *in vitro* enamel adhesion without detrimental effects of the aggressive PA gel with potential for remineralisation and saving time at the post-debonding step.

## Introduction

Malocclusion of teeth is common in different age groups (especially children and adolescents) and can have negative influence on dentofacial development, contributing to impaired orofacial function, patient’s psychological well-being and quality of life. A prevalence rate of orthodontic treatment needs of 21.3–39.5% has been reported in different European countries according to the official index for assessment of need for subsidized orthodontic treatment in a country of interest (Index of Orthodontic Treatment Need, IOTN)^1^. Orthodontic treatment using fixed appliances is widely used to correct irregular teeth and/or jaw discrepancies and improve aesthetic appearance of the face. Attachment of a fixed appliance involves bonding of metal or ceramic brackets onto the enamel surface of teeth and the use of special orthodontic wires/elastics to deliver forces to align the irregular teeth. The brackets are removed by the orthodontist using bracket debonding pliers at the completion of treatment (average treatment time,1–2 years). However, the treatment outcomes are frequently associated with a wide range of enamel damage including demineralisation or white spot formation that occur during the prolonged treatment, whilst enamel chipping or cracking very often ensues at the time of appliance removal due to the high debonding force required to remove the brackets^2^, which also results in some of the adhesive being left on the enamel surface. The removal of these adhesive remnants is required at the completion of treatment, which causes further trauma to the enamel^3^. These problems are commonly encountered with metal orthodontic brackets and have been exacerbated following the advent and popular use of ceramic brackets due to their rigidity and lack of deformation during removal, which has led to more frequent incidence of enamel damage^4^.

**Figure 1:**
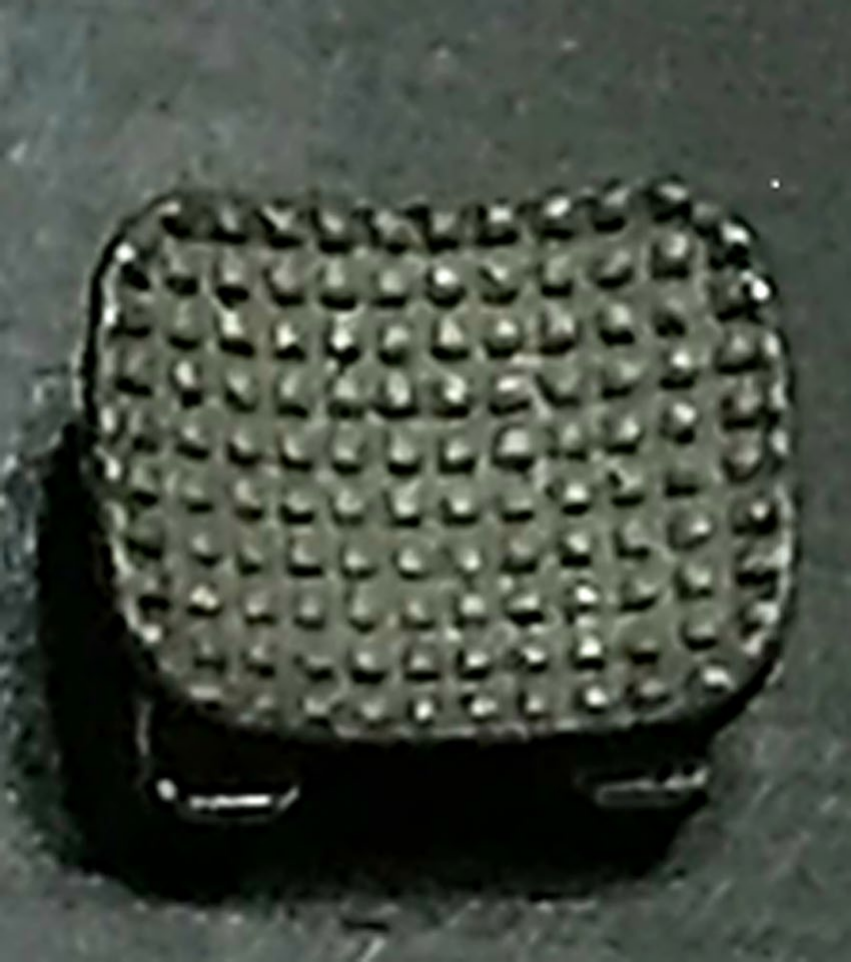
A metal bracket base with a mesh design containing numerous tiny undercuts to ensure maximum mechanical interlocking between the bracket and adhesive.

Fixation of brackets to teeth involves joining of two different solid substrates or adherents through an intervening adhesive layer. Bracket bases are currently provided with mesh designs that ensure robust mechanical interlocking between the bracket and adhesive (Fig. 1), whilst research is ongoing to improve enamel conditioning strategies that can attain clinically successful bond strengths yet maintain an unblemished enamel surface^5^. There is consensus that an etch-and-rinse approach using phosphoric acid remains the preferred choice for enamel conditioning, since it not only guarantees a durable bond to enamel but also seals and thus protects against degradation^6,7^. The tooth enamel surface is known for its low-energy and hydrophobic properties, with its outermost layer being rich in fluoride. Enamel etching with phosphoric acid (PA) removes the fluoride-rich layer, dissolves enamel mineral, creates micro-pores, and renders a high-energy and hydrophilic enamel surface to permit wetting by an adhesive; yet at the expense of increased surface porosity, susceptibility to staining, caries, more remnant adhesive and enamel loss^6,8^. Alternative techniques to the conventional acid-etching have been sought to decrease the adhesive remnants and enamel damage such as the use of self-etch adhesives and laser application, which result in acceptable bond strengths^9,10^. However, these methods are not able to eliminate the remnant adhesive left on enamel after bracket removal that exhibit comparable etch-patterns to the etch-and-rinse approach in terms of cracking and roughening of enamel surface; hence enamel damage is not eliminated^10–12^. On the other hand, methods to prevent and manage enamel demineralisation or promote remineralisation have been attempted through application of topical fluoride around orthodontic brackets^13^, incorporation of calcium-phosphates (CaP) in bonding resins for remineralisation^14^, and antibacterial agents in orthodontic cements^15^. However, these strategies only partially address the clinical concerns and although a slight regression of white spot lesion is reported^16^, improvements in terms of long-term ion release, adhesive remnants and/or enamel damage elimination are non-significant^16,17^.

In contrast to restorative dentistry where adhesive materials are generally bonded to teeth permanently, orthodontic treatment entails bonding of attachments (mostly brackets) for limited periods of time (1–2 years) to serve as means of application of force to teeth. Thus, enabling “in function” retention of both metal and ceramic brackets, minimising enamel demineralisation, and the ease of bracket debonding with minimal remnant adhesive and limited risk of permanent damage to the enamel surface are critical in orthodontics, yet challenging to achieve^18^. The overall goal of this study is to develop a novel enamel conditioning technique that aims to yield clinically acceptable bond strengths, induce CaP re-precipitation, and upon bracket removal leave no or minimal remnant adhesive on teeth and eliminate enamel damage. The rationale is premised on enamel etching exploiting a low-pH CaP paste capable of simultaneously dissolving surface enamel and precipitating a CaP phase on the enamel. The hypothesis is that the use of CaP precursors, which on mixing with an acid produces a CaP etchant capable of overcoming the detrimental effects of a strong acid alone on enamel damage, without compromising the bracket bond strength.

## Results

**Figure 2:**
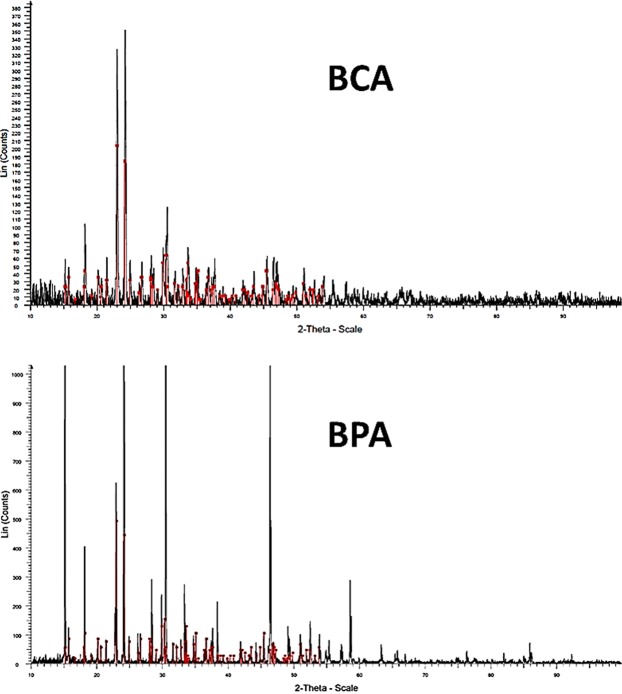
X-ray diffractograms of BCA and BPA powder samples. The red lines represent the main monocalcium phosphate monohydrate reference peaks [JCPDS file 00-009-0347 (I)] identifying a triclinic crystal system.

A commercial PA gel was used as the control group whilst the experimental etchants comprised of equimolar amounts of β-TCP and MCPM in presence of either phosphoric (BPA) or citric acid (BCA). The experimental etchants had a higher pH of 1.4 in comparison to the pH of the PA gel at 0.8. The X-ray diffraction (XRD) spectrum analyses indicated that monocalcium phosphate monohydrate (MCPM) is the predominant phase of the BCA and BPA formulations (Fig. 2), whose unit cell is triclinic with a lattice of 3 planes dimensions: a = 0.625 nm, b = 1.189 nm, c = 0.563 nm; and angles between the 3 planes: alpha = 96.67°, beta = 114.20° and gamma = 92.95°.

For both metal and ceramic brackets (Table 1), the control subgroup consistently exhibited the highest SBS mean value (p < 0.001), followed by lower values in the order BPA > BCA. However, on examination of the enamel surfaces post debonding of the brackets, the amounts of adhesive remnants in the control subgroup were significantly higher (p < 0.001) in comparison to the CaP subgroups, which interestingly left no or minimal adhesive residue without enamel damage (Table 2). Bracket debonding was also carried out after subjecting all the subgroups to 5000 thermo-cycles and it is noteworthy to mention that the BCA subgroup encountered 14 metal and 13 ceramic bracket failures during this procedure, hence only 6 metal and 7 ceramic brackets were tested for SBS. One-way ANOVA and Kruskal-Wallis tests exhibited statistically significant differences (*p* < 0.001) in SBS and ARI among the 3 subgroups (Tables 1 and 2, respectively).

**Table 1:** Shear bond strengths of metal and ceramic bracket debonding at 24 h and post 5000 thermo-cycles (between cold and hot water baths of 5 °C and 55 °C with a dwell time of 30 s in each bath and transfer time of 5 s).

Bracket Type	Sub groups	No. of Samples	Mean SBS (MPa)	SD	Median	Min.-Max.	ANOVA Statistics
Metal 24 h WS	Control	20	24.8 A*	5.8	24.6	13.4–34.9	*F* = 26.62 *df* = 2 *p* < 0.001
	BPA	20	18.2 B*	7.2	15.9	9.3–30.8	
	BCA	20	8.8 C*	3.0	8.6	4.5–14.2	
Ceramic 24 h WS	Control	20	32.1 A*	8.2	31.9	18.5–45.8	*F* = 26.77 *df* = 2 *p* < 0.001
	BPA	20	25.4 B*	7.5	25.7	13.5–35.8	
	BCA	20	12.5 C*	3.3	13.4	6.5–17.7	
Metal TC	Control	20	26.8 A*	4.2	26.7	17.4–34.5	*F* = 14.36 *df* = 2 *p* < 0.001
	BPA	20	18.5 B*	5.4	17.7	11.1–28.4	
	BCA	6 (14 BF)	1.8 C*	3.7	0.0	0.0–13.6	
Ceramic TC	Control	20	30.3 A*	7.3	32.3	17.5–46.5	*F* = 14.90 *df* = 2 *p* < 0.001
	BPA	20	20.8 B*	4.5	20.2	14.1–29.3	
	BCA	7 (13 BF)	3.0 C*	4.6	0.0	0.0–13.8	

A statistically significant difference was found among each three subgroups at the two time points.

BF: bracket failure, SBS: shear bond strength, TC: thermocycling, WS: water storage.

*Dissimilar letters indicate a statistically significant difference at *p* < 0.05 according to Tukey HSD post hoc multiple comparisons.

**Table 2:** Adhesive remnant index scores of metal and ceramic bracket debonding at 24 h and post 5000 thermo-cycles. The control subgroups showed significantly higher ARI scores than the two experimental subgroups, which exhibited non-significant differences between them.

Bracket Type	Sub groups	No. of Samples	ARI Scores				Statistics: Mann-Whitney	Statistics: Kruskal-Wallis
			0	1	2	3		
Metal 24 h WS	Control	20	2	6 (2EC)	6 (2EF)	6	A*	*H* = 49.69 *df* = 2 *p* < 0.001
	BPA	20	17	3	0	0	B*	
	BCA	20	20	0	0	0	B*	
Ceramic 24 h WS	Control	20	0	6	4 (3EC)	10 (4EF)	A*	*H* = 52.20 *df* = 2 *p* < 0.001
	BPA	20	13	7	0	0	B*	
	BCA	20	20	0	0	0	B*	
Metal TC	Control	20	3	7 (1EC)	6 (2EF)	4	A*	*H* = 53.30 *df* = 2 *p* < 0.001
	BPA	20	19	0	0	1	B*	
	BCA	6 (14 BF)	20	0	0	0	B*	
Ceramic TC	Control	20	0	1	5 (3EF)	14	A*	*H* = 57.30 *df* = 2 *p* < 0.001
	BPA	20	15	4	1	0	B*	
	BCA	7 (13 BF)	20	0	0	0	B*	

Adhesive Remnant Index (ARI) Scores: (0): No adhesive left on the tooth, (1): Less than half of the adhesive left on the tooth, (2): More than half of the adhesive left on the tooth, (3): All adhesive left on the tooth with a distinct impression of the bracket mesh. BF: bracket failure, EC: enamel crack, EF: enamel fracture, TC: thermocycling, WS: water storage.

*Dissimilar letters indicate a statistically significant difference at *p* <0.05 according to Mann-Whitney multiple comparisons.

Tukey HSD post hoc (for SBS) and Mann-Whitney (for ARI) multiple comparisons demonstrated that the control subgroups showed significantly higher mean SBS and ARI scores than the CaP subgroups, which exhibited non-significant differences between them in ARI scores only. The BPA subgroups exhibited significantly higher SBS than BCA subgroups.

**Figure 3:**
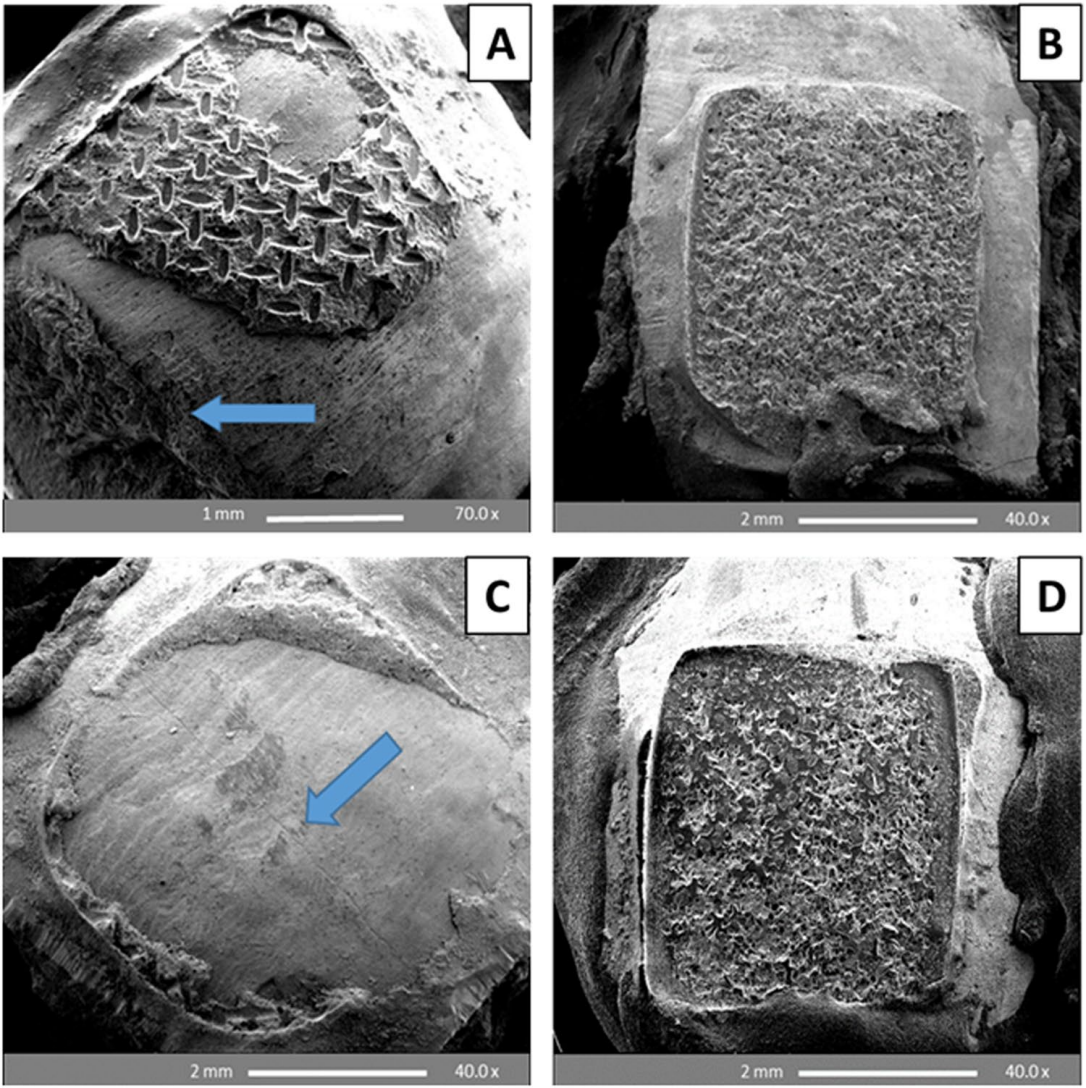
SEM images of premolar enamel surfaces etched with 37% phosphoric acid gel (30 s), and debonded after 24 h water storage (**A**,**B**) and thermocycling (**C**,**D**). A: Enamel chipping (arrow) with retention of adhesive (metal bracket debonding); B and D: Complete retention of adhesive (ceramic bracket debonding); C: Enamel cracking (arrow) with retention of adhesive (metal bracket debonding).

**Figure 4:**
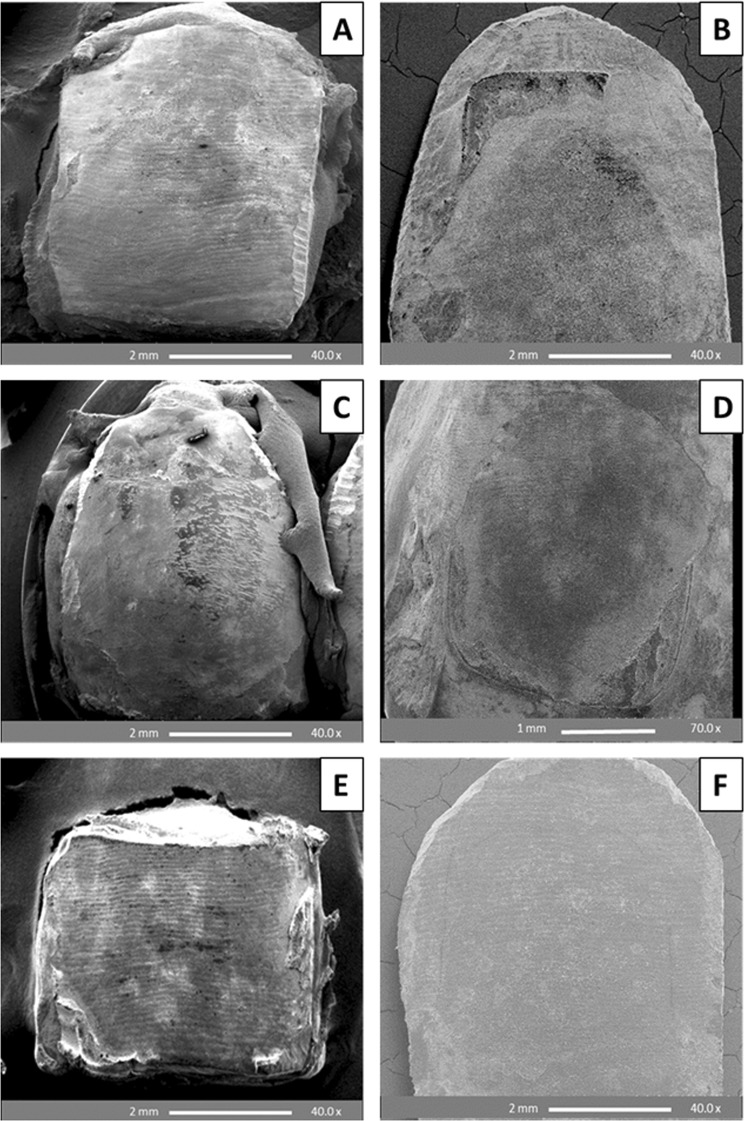
SEM images of premolar enamel surfaces showing unblemished enamel surfaces with no or minimal remnant adhesives following etching with BPA and BCA pastes (30 s). Bracket debonding was conducted after 24 h water storage (**A**,**B**,**E**) and thermocycling (**C**,**D**,**F**). (**A**–**D**): Treatment with BPA paste (A and C: metal; B and D: ceramic). E-F: Treatment with BCA paste (E: metal; F: ceramic).

The ARI scores obtained on enamel etching with PA gel mainly yielded scores of 2 and 3 for both metal and ceramic brackets. The SEM results showed retention of adhesive and enamel damage encountered following both 24 h water storage (WS) and thermocycling procedure (Fig. 3). In contrast, etching with BPA and BCA pastes yielded ARI scores of 0 and 1 for both metal and ceramic brackets, whilst maintaining smooth, unblemished surfaces with minimal evidence of enamel damage, and allowed perikymata preservation following both 24 h WS and thermocycling (Fig. 4).

Unlike the BCA, the CaP formulation made of 37% phosphoric acid (BPA) survived the rigorous artificial ageing tests, yielding satisfactory bond strengths to both metal and ceramic brackets, whilst upon bracket removal reducing the amount of remnant adhesive on teeth and eliminating the risk of enamel damage. Hence, the interaction of the BPA etchant paste with enamel surface was thoroughly investigated both before bracket bonding and after bracket removal.

### Enamel surface analysis post etching with BPA etchant (no bracket bonding) using confocal laser scanning microscopy (CLSM) and SEM

**Figure 5:**
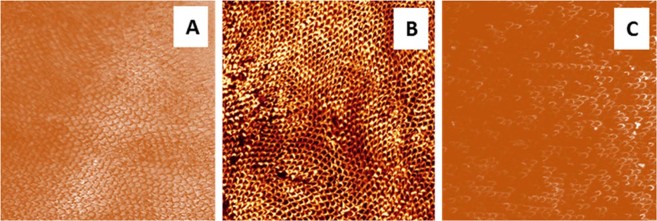
CLSM images (63x magnification) of highly polished flat buccal enamel surfaces. A: untreated surface showing the orientation of ground enamel prisms in various directions, B: Enamel etched with 37% PA gel exhibiting a uniform distribution of the honeycomb etch-pattern, C: Enamel etched with BPA paste showing an ill-defined etch-pattern with disperse clusters of micro-pores, perhaps incompletely covered by a re-precipitation layer.

Flat molar buccal surfaces were obtained by grinding and polishing, which clearly resulted in a scaly appearance of enamel prisms oriented in different directions (Fig. 5A). The etch-pattern produced on enamel etching with 37% PA gel yielded the typical etch-pattern (honeycomb appearance) due to the preferential dissolution of the enamel prism cores (Fig. 5B), whilst etching with BPA paste exhibited a less identifiable etch-pattern following similar etching time (30 s). However, production of diffuse honeycombs and disperse micro-pores is still evident and heralds the possible etching effect of the CaP paste (Fig. 5C).

**Figure 6:**
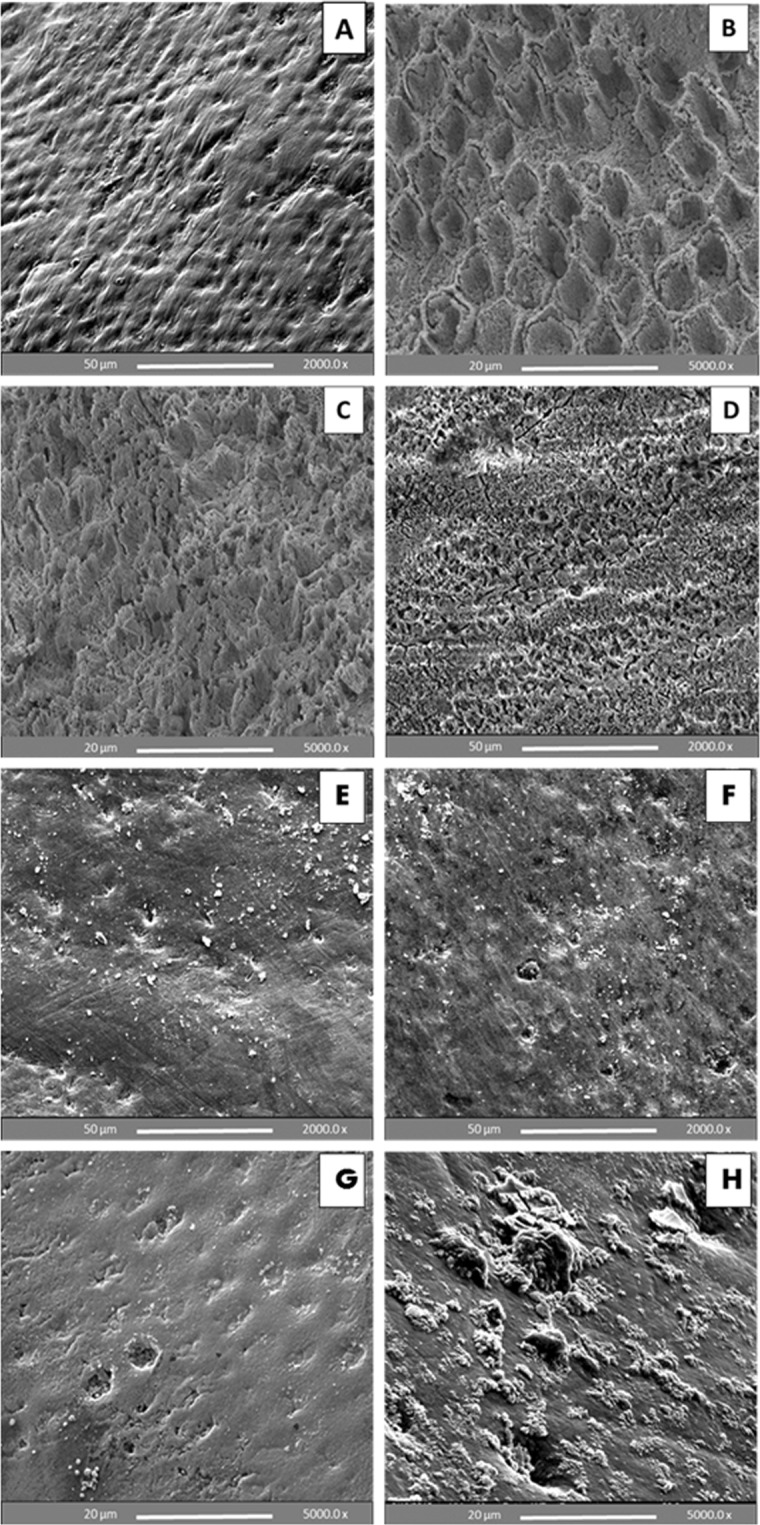
SEM images of un-bracketed premolar buccal enamel surfaces. (**A**) an intact surface showing irregular distributions of focal depressions, which represent various orientations of hydroxyapatite crystallites. (**B**–**D**) enamel surfaces etched with 37% PA gel. Etching quality varied from well-defined honeycomb areas where the prisms protruded with most of their cores being lost (**B**), or cobblestone areas where the prisms protruded with their peripheries being affected (**C**), to areas of both etch-patterns (**D**). Etching with BPA paste exhibited a milder etch-pattern, showing variations from an almost unaffected smooth surface (**E**) to a surface with small pits and/or openings accentuating the prismatic structures (**F**). CaP precipitates are evident, resulting in partial or complete obliteration of the micro-pores produced (**G** and **H**).

Figure (6A) shows an SEM image of a sound buccal enamel surface of a premolar tooth following pumice prophylaxis (10 s), depicting various orientations of enamel prisms running from the dentino-enamel junction towards the outer enamel surface with tiny surface scratches. According to the etch-pattern scale, enamel etching with 37% PA gel resulted in roughened surfaces featuring typical honeycomb and cobblestone etch patterns, i.e. types 1, 2 respectively, in addition to type 3 which is a mixture of type 1 and 2 etch-patterns (Fig. 6B–D). In contrast, etching with BPA paste demonstrated a mixture of types 4 and 5, with evidence of CaP precipitates (Fig. 6E–H). The CaP precipitations demonstrated a mixture of clustered grains, prismatic, spherical with lamellar-like morphology. Some enamel surface areas exposed to BPA paste etching showed variations that could not be distinguished from normal enamel anatomy, depicting zones of unaffected focal holes.

### SEM analysis of debonded premolar enamel surfaces after 24 h water storage and thermo-cycling

**Figure 7:**
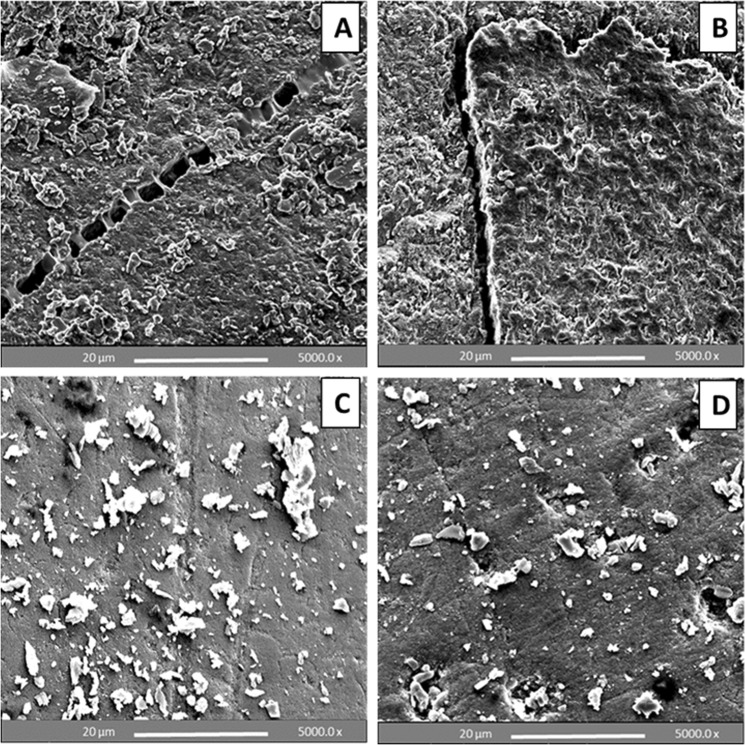
SEM images of de-bracketed premolar buccal enamel surfaces. Etching with 37% PA gel resulted in rough, irregular and cracked enamel surface with adhesive residues at metal (**A**) and ceramic (**B**) bracket debonding. Etching with BPA paste yielded smooth, regular surface showing CaP precipitants obliterating and surrounding the micro-pores with fine scattered scratches at metal (**C**) and ceramic (**D**) bracket debonding.

According to the enamel damage index (EDI), enamel etching with 37% PA gel yielded a mixture of grades 2 and 3 (generally rough surface with numerous coarse scratches or grooving) following both metal and ceramic bracket debonding (Fig. 7A,B). On the other hand, enamel etching with BPA paste yielded a mixture of grades 0 and 1 (generally smooth surface with fine scattered scratches) following both metal and ceramic bracket debonding. In contrast to 37% PA gel, BPA etchants left unblemished enamel surfaces with CaP re-precipitation. The outcomes consistently maintained a smooth enamel surface with minimal adhesive residues and minimal to no enamel damage, post metal and ceramic bracket debonding (Fig. 7C,D).

### Analysis of enamel decalcification using Raman spectroscopy

**Figure 8:**
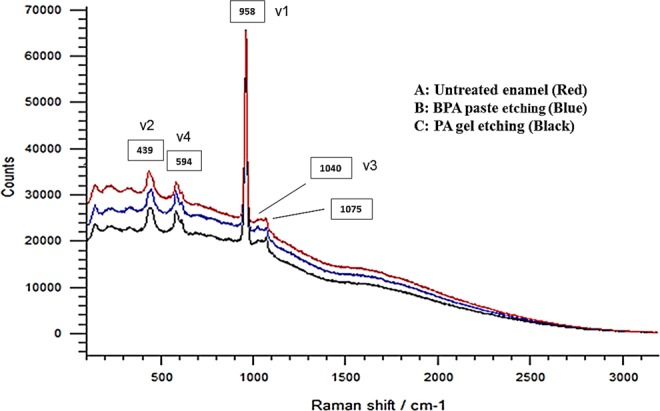
Raman spectra of intact enamel (**A**), enamel etched with BPA paste (**B**), and enamel etched with the control (**C**). The spectra identify 5 distinctive bands: four internal vibration modes of phosphate ion (ʋ1-PO at 958 cm^−1^, ʋ2-PO at 439 cm^−1^, ʋ3-PO at 1040 cm^−1^, and ʋ 4-PO at 594 cm^−1^), and B-type carbonate group at 1075 cm^−1^. Raman phosphate peak at 958 cm^−1^ characterises tetrahedral PO4 group (P–O bond) as the main and strongest phosphate peak within hydroxyapatite.

The flat-surface enamel specimens obtained from molar teeth were used to compare the Raman spectra of untreated and etched enamel surfaces. Each specimen was divided into three areas and used to record the spectra of untreated enamel area and two enamel areas conditioned with either of the two etching protocols (PA gel and BPA paste). All enamel surfaces showed the 5 characteristic peaks of enamel hydroxyapatite (HA) (Fig. 8): symmetric P-O stretching mode (*v*1-PO at 958 cm^−1^), doubly degenerate O-P-O bending modes (*v*2-PO at 439 cm^−1^), triply degenerate asymmetric P-O stretching modes (*v*3-PO at 1040 cm^−1^), triply degenerate modes of mainly O-P-O bending character (*v*4-PO at 594 cm^−1^), and the B-type carbonate at 1075 cm^−1^.

The four phosphate peaks were observed within untreated and treated enamel spectra with no difference in the Raman peaks; however, enamel conditioning with the two etching protocols resulted in marked reductions in the mean intensity of the 4 phosphate peaks as compared to those of untreated enamel area (Table 3). The strongest peak in sound and treated enamel spectra was that of ν 1 at 958 cm^−1^. The potential demineralisation/remineralisation effect induced by the two etching protocols was examined depending on the phosphate peak ratio (PPR) of *v*1/*v*4. The greatest drop in peaks intensity and *v*1/*v*4 PPR manifested in the 37% PA group, whilst the BPA group showed values closer to the untreated enamel. One-way ANOVA showed a statistically significant difference in *v*1/*v*4 PPR among the three groups (F = 10.11, df = 2, p < 0.001), whilst Tukey Post Hoc multiple comparisons revealed that 37% PA group had significantly lower PPR than the control (p < 0.001) and BPA (p = 0.010) groups, and a non-significant difference between untreated enamel and BPA group (p = 0.312).

**Table 3:** Raman peak intensities of 4 phosphate bands of buccal flat-surface enamel divided into 3 areas: intact enamel and treatments with 2 etchants. The mean ± SD of the 4 phosphate peaks and phosphate peak ratio (PPR) of v1/v4 were the highest in the intact enamel surface.

Groups	Peak Symbol	Peak Position	No. of Samples	Mean of Intensity	SD	Median	Min.-Max.
Intact Enamel	v2	439	10	2731.9	746.4	2848.0	1258–3884
	v4	594	10	389.8	24.2	392.5	353–437
	v1	958	10	5277.0	356.5	5324.5	4765–5791
	v3	1040	10	313.0	65.9	330.5	190–382
	v1/v4 PPR	—	10	13.6 A*	0.9	13.5	12.5–14.8
37% PA Gel	v2	439	10	1336.3	334.6	1322.0	628–1732
	v4	594	10	309.5	40.7	303.5	263–400
	v1	958	10	3378.0	297.0	3391.5	2995–3986
	v3	1040	10	129.0	26.2	136.5	74–154
	v1/v4 PPR	—	10	10.7 B*	1.0	11.2	8.65–12.3
BPA Paste	v2	439	10	1885.2	369.0	2031.0	1004–2170
	v4	594	10	300.2	36.6	315.0	223–333
	v1	958	10	3849.3	421.4	3897.5	3289–4449
	v3	1040	10	205.6	46.5	221.0	120–252
	v1/v4 PPR	—	10	12.9 A*	1.0	12.7	11.0–15.2

Depending on the PPR of v1/v4, enamel etching with BPA paste resulted in less demineralisation than etching with 37% PA, with a probable remineralisation effect.

*Dissimilar letters indicate a statistically significant difference at *p* < 0.05.

## Discussion

Orthodontic treatment is aimed to align mal-positioned teeth and correct malocclusions. In order to enable movement of teeth, force is applied through use of bonding attachments (mostly brackets) that are bonded to enamel. It is a very successful treatment, however, alterations to the enamel surface occur during and at the bracket removal stage, which are induced by the iatrogenic manipulation. Enamel acid-etching is one of the most fundamental methods of facilitating adhesion and the acid acts by decalcification that causes an irregularity on the surface of enamel. Phosphoric acid liquid or gel is widely used to etch enamel, and conventional bracket bonding procedure is based on enamel pre-conditioning with PA gel for 30 s, water rinsing of the gel, then air-drying the etched surface before attaching the bracket using resin adhesives^5^. This new bioactive orthodontic etchant can alleviate subsurface enamel demineralisation in comparison with the traditional phosphoric acid etchant and the pre-clinical viability assessment using *in vitro* studies show exemplary advantages. The new low-pH CaP paste etchant application is a clinically viable bracket bonding method with steps similar to PA application^7^ that yielded adequate bond strength with the advantages of minimal adhesive remnants and no enamel chipping or cracking on debonding of the bracket. The mechanism of bonding to enamel in the conventional etch-and-rinse approach is essentially based on the loss of superficial enamel and preferential dissolution of the underlying enamel on phosphoric acid treatment (decalcification) that create asperities on the surface to enable ingress of resin monomers that upon polymerisation become micro-mechanically interlocked^5^. Etching enamel with phosphoric acid leads to dissolution of the enamel hydroxyapatite releasing calcium, which to a small extent is able to buffer if the enamel surface is not agitated by resisting changes in hydrogen ion concentration. However, the inclusion of a low pH calcium phosphate in the etchant becomes more effective in buffering, limiting enamel decalcification and, instead, enhancing CaP re-precipitation without adversely affecting the etching process.

Although there is no general consensus on the optimal bond strength required for successful clinical performance of bonded brackets, a minimum range of 6–10 MPa has been suggested as suitable for bracket bonding to achieve acceptable clinical performance^19–21^ and remains the standard reference value for the vast majority of *in vitro* bond strength studies^22,23^. On the other hand, adhesion forces should not be too strong in order to avoid enamel loss after debonding (40–50 MPa)^24^. Therefore, the ideal orthodontic biomaterial should have bonding forces included in the interval of 5–50 MPa, even if these limits are mostly theoretical^25^. Literature findings on enamel etched with 37% PA using the conventional etch-and-rinse approach report an average bracket bond strength ranging from 9–35 MPa, with usually higher values attained for ceramic than metal brackets^7^. The SBS results of most metal and ceramic subgroups in this study are within the confines of this range. Enamel etching with conventional 37% PA gel (pH = 0.8) before metal or ceramic bracket bonding resulted in significantly higher mean SBS than etching with the CaP pastes prepared from comparably strong acids. Mixing of CaP powders with 37% PA and 5 M CA imposed a buffering effect by raising the pH of these acids in the resulting paste from 0.8 to 1.4 and from 1.0 to 1.4 respectively, hence yielding less aggressive etchants. Typically, when equimolar amounts of β-TCP and MCPM are mixed in aqueous conditions, brushite forming cements are formed; however, different end-products, such as stoichiometric or calcium-deficient hydroxyapatite, calcium octaphosphate or brushite may result by an acid-base or hydrolysis reaction, depending on the pH^26^. In addition, acid-etching of enamel creates surface micro-pores by dissolving calcium and phosphate ions, which re-precipitate as brushite or MCPM depending on the type and concentration of the etchant used. Formation of brushite has been reported to follow enamel conditioning with PA of concentrations less than 27%, whilst MCPM is formed when using concentrations exceeding 27%^27,28^. The latter finding is supported in this study as the XRD data revealed MCPM as the predominant phase of the BPA paste (made of β-TCP and MCPM powders in equimolar amount) prepared by mixing in presence of 37% PA. The milder etching effect and diminished resin infiltration due to the CaP salt precipitation led to the lower SBS values attained by the CaP BPA etchant compared to the PA gel; however, the mean SBS obtained from BPA paste subgroups ranged between 18.2–25.4 MPa, which were well above the lower limit for acceptable clinical performance. The stagnation of CaP crystals and resistance to water irrigation due to the low solubility and the volume of crystallites^29^ remain the main factors that impede the complete infiltration of the resin into the micro-pores, hence adversely affect the bond strength.

The citric acid based etchant (BCA) pastes, however, were unable to sustain the thermocycling regime and resulted in statistically significant low SBS values. Despite the use of a high CA concentration (5 M, pH = 1.0) to prepare BCA pastes, the buffering effect induced by mixing with the CaP powders led to an enamel-adhesive interface of inadequate resistance to prolonged thermal fluctuations as indicated by all failures at the interface. A finding in literature which supports this experimental observation, albeit for a different application, is that the addition of calcium to CA minimised enamel erosion and reduced its erosive and demineralisation effects significantly^30,31^.

Etching of enamel with 37% PA generates high bracket bond strengths, however this excessive bond strength has been a point of contention as debonding these brackets often lead to enamel cracks and fractures^32^. The debonding of the brackets also tend to leave adhesive remnants on the enamel surface, hence enamel clean-up and polishing is required to remove them (usually using rotary burs) resulting in increased chair time and inevitable enamel scratching^10,33^. The results of the adhesive remnant index when enamel etching was carried out with 37% PA gel were in agreement with previously reported data and as expected resulted in significantly greater amount of remnant adhesives after bracket removal with six samples exhibiting cracked enamel and 11 with chipping. In contrast, the application of CaP etchant pastes to effect bonding left minimal or no adhesive with no signs of enamel damage. Thus far no study or other bonding systems have been able to achieve this level of unperturbed enamel surface without significantly compromising the bond strength. The unprecedented combined advantages of this approach are expected to impact orthodontic treatment leading to less enamel damage, thereby alleviating long term problems associated with it. The ARI scores depend on the interplay of many factors including the bracket base design, etching technique, adhesive type, and location of the debonding force; therefore, higher ARI scores are not necessarily only concomitant with higher bond strengths^34,35^. However, this means that attaining bond strengths exceeding the minimum requirement for clinical performance whilst maintaining minimal remnant adhesive on the enamel post bracket debonding is still possible. Manipulating the etching chemistry to weaken the enamel-adhesive interface was achieved in this study through the employment of acidic CaP pastes with potential enhancement of CaP precipitation, hence facilitating the preferable debonding failure at enamel-adhesive interface and saving chair-side time and cost at the post debonding clean-up stage.

Removal of the outermost enamel layer, prior to studying enamel mineral changes, reduces the aprismatic enamel, if any, as well as the variation between the biological samples^36,37^. To examine the enamel surfaces after treatment with the control and the experimental etchant paste, characterisation of the resulting surfaces by different techniques is essential; hence flat enamel specimens were used to enable CLSM and Raman spectroscopy analyses. The CLSM can provide evidence about the enamel etching effects induced by acids and the enamel remineralisation potential with remineralising agents depending on the degree of image fluorescence, which increases with the greater loss of organic matrix and exposure of enamel prisms^8,38^. This is achieved through the use of fluorescent dyes (e.g. Rhodamine B dye) which promote certain emission wavelengths when they are excited by laser with specific wavelengths, hence can be traced at their locations in a material and/or tissue at dilute concentrations^39,40^. The CLSM images revealed that the etched surfaces showed a clearly visible increase in fluorescence, evidenced by the more apparent honeycomb morphology in areas of enamel etched with 37% PA gel, the etched enamel prisms clearly exposed and a better defined honeycomb morphology with an apparently greater loss of organic matrix (Fig. 5B). In contrast, etching with the BPA paste demonstrated an ill-defined pattern with disperse islands of micro-pores (Fig. 5C). The milder, less discernible etch-patterns produced by the etchant pastes may be traced back to their higher pH (1.4) than that of 37% PA gel (0.8), and the enhanced CaP precipitation on the etched enamel surface. The CaP precipitation was confirmed by SEM imaging conducted both before bonding and after bracket debonding.

Before bonding, the SEM images revealed that treatment with BPA paste yielded the gentle etch-patterns similar to observations using CLSM and resulted in CaP precipitants, which tended to aggregate and occlude the micro-pores produced by the etching effect (Fig. 6E–H). Further to partial or complete obliteration of the micro-pores, the CaP crystals tended also to reside in etched, irregular and rough surface areas helping to regularize and locally flatten the etched enamel surface; thus contributing to surface wettability and bond strength. After bracket debonding, the SEM images of enamel etched with the BPA pastes demonstrated a relatively smooth, flat surface rich in stagnant CaP particles with minimal adhesive remains and no evidence of enamel cracks or fractures (Fig. 7C,D) as compared to an irregular, rough and cracked surface with lots of remnant adhesive produced by the control PA gel (Fig. 7A,B). Breakage or crushing, induced by the bracket debonding force, of the precipitated and/or partially or completely penetrated CaP precipitants inside the micro-pores may account for their appearance on the enamel surface after debonding. Many SEM studies have reported enamel surface irregularities, roughening, grooving and cracking following metal^41–43^ and ceramic bracket debonding^44,45^. However, no damaging effects to the enamel were seen following bracket removal after etching with the BPA pastes in this study, with a majority of the teeth showing adhesive failure at the enamel leaving almost unblemished surfaces. This can be attributed to the weakening effect induced by CaP particles penetration into the porous enamel and precipitation at the enamel-adhesive joint facilitating a safer bracket debonding at this interface.

Micro-Raman spectroscopy is used both as a quantitative and qualitative chemical assessment methodology for biological samples. In this study, the Raman spectra of all untreated and etched enamel surfaces demonstrated the 5 characteristic peaks of enamel similar to previous findings^46–48^; however, etched enamel yielded lower phosphate peak intensities than untreated enamel. The Raman phosphate peak at 958 cm^−1^ is characteristic of the tetrahedral PO_4_ group (P–O bond) as the main and strongest phosphate peak within hydroxyapatite^48,49^. Since Raman peak intensity is proportional to the number of molecules within the volume of the scanned area^50^, monitoring the intensity of this peak has been used to assess the degree of demineralisation within carious enamel^51,52^. Furthermore, it has been recently used to assess potential increase in phosphate content within white spot lesions following remineralisation treatments using bioactive glass powders^53^, and to examine the remineralisation potential of artificial carious lesions with high-frequency microwave energy therapy^54^. Following a similar principle, a comparison of the 4 phosphate peaks between the untreated and etched enamel showed that areas etched with 37% PA gel or the experimental BPA paste exhibited marked reductions in their intensities, with the main phosphate peak (*v*1) showing the greatest, and peak *v*4 showing the lowest decrease in intensity (Table 3). Interestingly, the latter peak, as compared to peaks *v*2 and *v*3, consistently yielded the lowest variance in the two treatment areas; in other words, it was the only peak that consistently showed a standard deviation lower than 25% of the mean value in both treated areas. Moreover, this peak (v4) and the strong peak *v*1, were found to be distinctive for HA and reliable in differentiating between HA and other calcium phosphates^46,55^. Accordingly, to avoid the potential of biased demineralisation/remineralisation information solely extrapolated from the main phosphate peak (*v*1), peak *v*4 was used as a reference in this study to calculate the *v*1/*v*4 phosphate peak ratio (PPR). The outcomes of PPR comparisons confirmed the remineralisation potential following enamel etching with BPA paste, exhibiting a non-significant difference in comparison with the untreated enamel group and a significantly higher PPR than enamel with the conventional 37% PA gel etching. The PPR of BPA group was close to that of untreated enamel, confirming the SEM findings in terms of both the mild etch pattern produced and the considerable CaP precipitation. To enable the efficacy of a bonding system, an ideal scenario is an accurate simulation of the oral environment, however an extremely difficult goal compounded by patient related variables. It is also extremely difficult to clinically distinguish the adhesive potential of a specific bonding system independent of the numerous variables that can influence either the quality or the longevity of bracket bonding to enamel^22,23^. However, mimicking the extreme conditions that may be experienced in the oral environment can reflect on clinical performance. In addition, *in vitro* investigations allow for more standardized procedures for testing a specific bonding system and are valuable for at least the initial screening and selection of materials. The outcomes of this study serve as an *in vitro* robust screening of the orthodontic etchant system towards a controlled clinical investigation as the results obtained following rigorous testing are promising for a successful *in vivo* performance. Other variables that have been demonstrated to have significant effects on shear bond strength values and ARI scores of dental and orthodontic materials are the etching period and type of adhesive used and enamel contamination, however the present study reports the result of a systematic *in vitro* study using one bonding agent with a specified etching period and a specific enamel cleaning regimen. Hence the results may change if a different bonding agent is used but the efficacy of the etchant has been clearly established with the recommended enamel treatment.

## Conclusion

In contrast to the conventional paradigm of incorporating remineralising agents in the adhesives to release calcium and phosphate ions, which only partially address the clinical concerns with non-significant enamel damage limitation, our strategy of using a two-in-one acidic CaP etchant paste successfully resulted in less aggressive etching of the enamel and creating a “CaP-built-in” enamel surface simultaneously. This new strategy of enhanced CaP re-precipitation promotes safer enamel etching and bracket debonding and may serve as an adjunct to contemporary attempts of enhancing enamel resistance to demineralisation during orthodontic treatment.

## Materials and Methods

Extracted human premolar and molar teeth were collected from adolescent and young patients (10–25 years) to carry out *in vitro* bonding procedures after acquiring ethical approval from the National Research Ethics Service Committee London-Riverside (REC Reference 14/LO/0123). Informed consent for study participation was obtained from each participant, and from a parent and/or legal guardian for participants under the age of 18 years and all methods were performed in accordance with the relevant guidelines and regulations. After extraction, the teeth were cleaned in running water to remove any blood and soft tissue debris, then stored in a 1% chloramine-T trihydrate bacteriostatic/bactericidal solution for a maximum of one week and thereafter stored in distilled water (ISO/TS 11405:2015)^56^. Criteria for tooth selection included: intact buccal enamel surface without cracks and caries (examined under stereomicroscope x10 magnification), with no history of previous endodontic, orthodontic or bleaching treatments^57^.

Beta-tricalcium phosphate (β-TCP) and monocalcium phosphate monohydrate (MCPM) powders (Sigma-Aldrich, UK) were used in equimolar amounts as principal constituents of the solid phase for preparing acidic CaP etchant pastes (capable of forming brushite on enamel) using 37% PA [BPA] and 5 M citric acid (CA; Acros Organics™, Fisher Scientific, UK) [BCA] as a liquid phase, in a powder-to-liquid ratio of 0.8:1 and 3:1 respectively. The assigned powder and liquid were mixed using a stainless steel spatula on a glass slab for 30 s until a homogenous workable paste was obtained. The 30 s pH of the resulting paste was measured by a flat-end electrode using a digital pH meter (Oakton, Singapore). All formulations were prepared under ambient conditions (20–23 °C and 50–60% humidity). A conventional 37% PA gel (Orthotechnology, USA) was used as the control etchant, whilst Transbond XT light-cure primer and adhesive (3 M Unitek, Monrovia, California, USA) were used for all brackets bonding. Two types of pre-adjusted upper premolar brackets were used: metal (Pinnacle, stainless steel, MBT, slot 0.022 × 0.028 inch, Orthotechnology, USA) and ceramic (NeoCrystal, monocrystalline sapphire, MBT, slot 0.022 × 0.028 inch, Henry Schein, USA).

### Sample preparation for bracket bonding

Premolar teeth were mounted in acrylic blocks using rubber moulds (14 mm length ×14 mm width ×17 mm depth). First, soft sticky wax was used at the tooth root apex inside the mould to align the tooth in such a way that the middle third of the buccal surface was parallel to the analyzing rod of a surveyor, to ensure a debonding force running parallel to the bonded bracket base. Then self-cure clear acrylic (Oracryl, Bracon, UK) was poured around the tooth up to about 1 mm apical to the level of cemento-enamel junction. The teeth were subsequently stored in distilled water at lab temperature until bonding.

### Enamel conditioning and bracket bonding procedure

The mounted teeth were randomly allocated into two main groups to be bonded with 120 metal (stainless steel) and 120 ceramic (sapphire) brackets. Each main group was further divided into three subgroups according to the type of etchant used: 37% PA gel [control, C] (Resilience, Ortho Technology, Inc. Tampa, Florida, USA), CaP paste made of 37% PA solution [BPA], and CaP paste made of 5 M CA [BCA]. The conventional etch-and-rinse protocol^5^ was followed to bond all brackets. The buccal surface of each tooth was polished (10 s) with pumice slurry using rotary rubber cups, followed by water irrigation (10 s) and oil-free air dryness (10 s). This was followed by application of the etchant on the middle third of the buccal surface for 30 s, then irrigation with water for 20 s and dryness for 20 s. A thin layer of bonding primer was applied onto the etched enamel and spread by air-jet (3 s). The bracket base was loaded with the adhesive composite and placed onto the middle third of buccal surface with a pressing force of 300 g (Correx force gauge, Bern, Switzerland) for 10 seconds to ensure an even adhesive thickness. Excess composite was removed by a dental probe. LED Light curing (3 M ESPE, Elipar DeepCure-S, USA, 1470 mW/cm^2^ light intensity) was applied for 20 s (10 s on each mesial and distal side) according to the manufacturer instructions.

Following bracket bonding, the bonded teeth were stored in distilled water at 37 °C for 24 h. Then half of the samples (n = 20 per subgroup) were debonded, while the other half subjected to a thermo-cycling regimen of 5000 cycles between cold and hot water baths of 5 °C and 55 °C with a dwell time of 30 s in each bath and transfer time of 5 s (ISO/TS 11405:2015, testing of adhesion to tooth structure), followed by bracket debonding.

### Bracket debonding for SBS, Adhesive Remnant Index (ARI) and enamel damage assessment

The SBS test was conducted using a chisel on a universal testing machine (Instron, Model 5569 A, USA) with an occluso-gingival load applied vertically at the bracket base at a crosshead speed^14^ of 0.5 mm/min and SBS values were calculated in MPa by dividing the load at failure by the bracket base surface area. The debonded teeth were examined under x10 magnification (Stereomicroscope (MEIJI, EMZ-TR, Japan) for the amount of remnant adhesive left and scored according to the ARI system^58^. Enamel damage was also examined under x10 magnification, and two random samples of each subgroup were further sputter-coated with gold palladium (15 nm) and examined using SEM machine (Hitachi High Technologies, S-3500N) at an accelerating voltage of 10 kV.

### Sample preparation for X-Ray diffraction (XRD)

Calcium phosphate phase determination of BCA and BPA formulations was carried out using XRD. Three pastes of each formulation were prepared, each was kept in a glass vial for 24 h and ground with a mortar and pestle after complete setting to get CaP powder. The powder samples were examined using a Bruker D4 Diffractometer in a Flat-plate geometry using Cu Kα12, 40 kV and 30 mA X-ray radiation.

### Scanning Electron Microscopy (SEM) of enamel before bonding and after bracket debonding

In order to examine the etch-patterns produced, CaP precipitation potential and enamel damage, SEM examination of the etched buccal enamel surface with 37% PA gel and BPA paste was conducted before bracket bonding and after bracket removal.

### SEM analysis of enamel before bonding

Nine extracted premolars were used in this analysis and divided into three groups: three teeth were kept intact and the others randomly etched with either of the two aforementioned etchants. The crown of each tooth was sectioned mesio-distally through the occlusal central fossae using a diamond wafering blade to obtain nine buccal halves. The etchant was applied on the buccal enamel surface for 30 s, followed by irrigation with water for 20 s and then dried for 20 s. All samples were kept dry at ambient laboratory conditions for 24 h, then sputter-coated with gold palladium (15 nm) and examined using SEM machine (Hitachi High Technologies, S-3500N) at an accelerating voltage of 10 kV.

The quality of enamel etch pattern produced was assessed and scored according to the etch-pattern scale^59^:

Type (1): Ideal etch, preferential dissolution of the prism cores resulting in a honeycomb-like appearance.

Type (2): Ideal etch, preferential dissolution of the prism peripheries resulting in a reverse honeycomb or cobblestone-like appearance.

Type (3): A mixture of type 1 and 2 patterns.

Type (4): Pitted, roughened enamel surface. Structures look like unfinished maps or networks, enamel prisms not evident.

Type (5): Flat smooth surface, no apparent etch.

### SEM analysis of enamel post debonding of brackets

The samples for this analysis were obtained from bracket-bonded premolars previously subjected to metal and ceramic bracket debonding following 24 h water storage at 37 °C and 5000 cycles thermo-cycling. Three metal and three ceramic bracket-debonded premolars of each of the PA gel- and BPA paste-etched subgroups were randomly collected, the crown of each tooth was sectioned mesio-distally through the occlusal central fossae using a diamond wafering blade to obtain the buccal bracket-debonded half, sputter-coated with gold palladium and examined with SEM. The debonded enamel surface was assessed according to the Enamel Damage Index^60^ which includes the following categories:

Grade (0): Smooth surface without scratches, and perikymata might be visible.

Grade (1): Acceptable surface, with fine scattered scratches.

Grade (2): Rough surface, with numerous coarse scratches or slight grooves visible.

Grade (3): Surface with coarse scratches, wide grooves, and enamel damage visible to the naked eye.

### Flat-surface enamel specimen preparation for Confocal Laser Scanning Microscopy (CLSM) and raman spectroscopy

**Figure 9:**
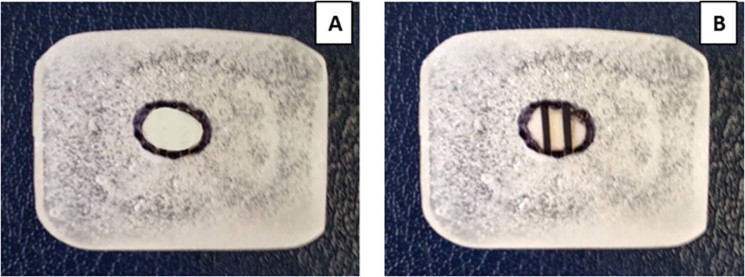
A representative image of a flat, highly polished molar buccal enamel surface (circled) embedded in an acrylic block (**A**), and divided into 3 zones: one untreated enamel and two etched with 37% PA gel or BPA paste before examination (**B**).

The buccal crown half of each molar tooth was sectioned mesio-distally through the occlusal central fossae using a diamond wafering blade (XL 12205, Extec Ltd., UK). The buccal enamel halves were mounted face down in cold-cure acrylic resin using silicon molds (8 × 21 × 24 mm). The outer enamel layer was removed using a water-cooled rotating polishing machine (Meta-Serv 3000 Grinder-Polisher, Buehler, Lake Bluff, Illinois, USA) and a sequential polishing protocol with silicon carbide abrasive disks (Versocit, Struers A/S, Copenhagen, Denmark) at a speed of 200 rpm: 600-grit for 10 s, 1200-grit for 20 s, 2500-grit for 30 s, and 4000-grit for 60 s. Each was followed by 1 min of water bath ultra-sonication to remove the smear layer at the enamel surface, excepting the 4000-grit which was followed by 3 min ultra-sonication. This standardized polishing protocol permits the removal of approximately 400 µm from the outer enamel layer and results in a flat, smooth and highly polished enamel surface^36^ (Fig. 9A). The surface of each enamel sample was divided into 3 areas using adhesive tapes: one untreated enamel, and the other two etched with either 37% PA gel or BPA paste according to the aforementioned etch-and-rinse protocol used in the bracket bonding procedure. This was followed by replacing the adhesive tapes with a black permanent marker line (Fig. 9B). The samples were kept dry at ambient laboratory conditions for 24 h before examination.

### Confocal Laser Scanning Microscopy (CLSM) samples

CLSM was conducted to compare the etch-patterns produced by the BPA etchant paste and 37% PA gel. Before enamel etching, 0.1 wt.% Rhodamine B dye^61^ (Sigma–Aldrich, UK) was added to the control 37% PA gel and the liquid phase (37% PA solution) of the BPA paste to obtain fluorescent etchants. Three samples were used for CLSM examination. The microscopy examination was performed using a CLSM (Leica SP2 CLSM; Leica, Heidelberg, Germany) equipped with a 63x magnification/1.4 NA (numerical aperture) oil-immersion lens, and a laser illumination setting of 568-nm krypton (rhodamine excitation). Representative images of the most common distinguishing characteristics detected in each specimen were captured. All images were further reconstructed with Image J software.

### Raman spectroscopy samples

Ten enamel samples were used. A Renishaw inVia Raman microscope (Renishaw Plc, Wotton-under-Edge, UK) running in a Streamline scanning mode was used to scan each area using a 785-nm diode laser (100% laser power) focused using a 20×/0.45 NA air objective. The signal was acquired using a 600 lines/mm diffraction grating centered at 750 cm^−1^ and a CCD (charge-coupled device) exposure time of 2 s. The microscope was calibrated using an internal silicon sample with a characteristic band at 520 cm^−1^. For each control and treated area, a Raman map was recorded at the middle part covering an area of 200 × 300 µm^2^ acquired with a 2.7 µm resolution. Raman maps were exported into an in-house curve-fitting software to fit the spectra and obtain the intensity mean of four phosphate peaks, in addition to the phosphate peak ratio (v1/v4)^53^.

### Statistical methods

Sample size determination was based on one-way analysis of variance (ANOVA) comparing the SBS of three different subgroups. A sample of 15 specimens per subgroup is required to detect a significant difference with an effect size of 0.35 and 80% power 2-tailed test at 5% level of significance. Sample size calculation was achieved using G-power version 3.1.7 (Franz Faul, Uni Kiel, Germany). Analysis was conducted using SPSS statistical software (version 22, SPSS Inc., IBM, Chicago, USA). Data were tested for normality using Histogram/Q-Q plots/Shapiro-Wilk tests. One-way ANOVA was conducted for parametric data analysis (SBS, Raman peak intensity), followed by Tukey HSD post hoc multiple comparisons. Kruskal-Wallis and Mann-Whitney tests were carried out for non-parametric data analysis (ARI score). All statistical analyses were conducted at level of significance *p*< 0.05. The statistic value of level of significance for multiple Mann-Whitney comparisons, when conducted after Kruskal-Wallis test, was 0.008 instead of 0.05; calculated according to Bonferroni correction.
